# Dispersion of Nanomaterials in Aqueous Media: Towards Protocol Optimization

**DOI:** 10.3791/56074

**Published:** 2017-12-25

**Authors:** Inder Kaur, Laura-Jayne Ellis, Isabella Romer, Ratna Tantra, Marie Carriere, Soline Allard, Martine Mayne-L'Hermite, Caterina Minelli, Wolfgang Unger, Annegret Potthoff, Steffi Rades, Eugenia Valsami-Jones

**Affiliations:** ^1^School of Geography, Earth and Environmental Sciences, University of Birmingham; ^2^Analytical Science, National Physical Laboratory; ^3^INAC-LCIB, Université Grenoble Alpes; ^4^CEA, INAC-SyMMES; ^5^NIMBE, CEA, CNRS, Université Paris-Saclay, CEA Saclay; ^6^Chemical, Medical and Environmental Science, National Physical Laboratory; ^7^BAM Division 6.1 'Surface Analysis and Interfacial Chemistry', BAM Federal Institute for Materials Research and Testing; ^8^Fraunhofer Institute for Ceramic Technologies and Systems

**Keywords:** Environmental Sciences, Issue 130, Nanomaterials, dispersion, sonication, characterization, protocol optimization, optimization

## Abstract

The sonication process is commonly used for de-agglomerating and dispersing nanomaterials in aqueous based media, necessary to improve homogeneity and stability of the suspension. In this study, a systematic step-wise approach is carried out to identify optimal sonication conditions in order to achieve a stable dispersion. This approach has been adopted and shown to be suitable for several nanomaterials (cerium oxide, zinc oxide, and carbon nanotubes) dispersed in deionized (DI) water. However, with any change in either the nanomaterial type or dispersing medium, there needs to be optimization of the basic protocol by adjusting various factors such as sonication time, power, and sonicator type as well as temperature rise during the process. The approach records the dispersion process in detail. This is necessary to identify the time points as well as other above-mentioned conditions during the sonication process in which there may be undesirable changes, such as damage to the particle surface thus affecting surface properties. Our goal is to offer a harmonized approach that can control the quality of the final, produced dispersion. Such a guideline is instrumental in ensuring dispersion quality repeatability in the nanoscience community, particularly in the field of nanotoxicology.

**Figure:**
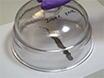


## Introduction

Sonication is the process of generating cavitations, which involves the creation, growth, and collapse of bubbles (often called hot spots) formed in liquid due to the irradiation of high intensity ultrasound^1^. In a laboratory setting, the sonication method is carried out using a sonicator. There are different sonicators, all having the general function of de-agglomerating particles, which disperse in a liquid medium as individual (or primary) particles. By applying sonication, sample homogeneity can improve, potentially achieving a much narrower particle size distribution. An important aspect to consider in the dispersion process is stability of the final dispersion. Here, the stability of the suspension is defined as where the particles do not settle or sediment down in their dispersed state and the average hydrodynamic diameter measurements do not vary by more than 10% between the five repeated measurements taken during that time (around 10 min)^2^,^3^. There are several ways to measure dispersion stability. This includes estimation of zeta potential (ZP) through measurement of electrophoretic mobility of particles. Another is to measure the characteristic absorption of nanoparticles in the UV spectral range^4^.

In the field of nanotoxicology, the ability to have control over dispersion quality is very important, as the dispersion step will determine key physicochemical properties, such as particle size/size distribution, shape, aggregation/agglomeration, surface charge, *etc.* This in turn will ultimately affect the interaction of particles with test media and outcome of various *in vitro* and *in vivo* experiments, in order to deduce the potential hazards of nanomaterials.

Sonication is commonly carried out by either using a probe-type (direct) or an ultrasonic bath, or ultrasonic probe with a vial tweeter (indirect sonication). All types of sonication are available in a range of intensity and output power settings, sometimes adapted with a different type of sonotrode for specific processes or requirements, and are suitable for liquid volumes ranging from 2 to 250 mL. Although probe ultrasonication is known to perform better than bath sonication because of high localized intensity^5^, bath sonication is often preferred over probe-type for the preparation of toxicological test suspensions because of the possible contamination risk through the tip, erosion of titanium probe tip after prolonged use, and probe immersion depth discrepancies. Similarly, an ultrasonic probe fitted with a vial tweeter is advantageous over the direct probe due to the above-mentioned contamination risks as well as the operation friendliness of the equipment. Several vials are sonicated at the same time and at the same intensity. This not only saves time but ensures that all the samples are treated equally, which makes the results among samples more reliable and comparable. In the safety research of nanomaterials, contamination is always avoided. However, the probe sonicator does not fit this requirement and has not been tested. Probe sonicators are known to cause some unavoidable side effects such as sample contamination due to tip erosion as well as reduced energy output leading to alterations of dispersion conditions, hence compromising data reproducibility^6^,^7^,^8^. Moreover, samples are usually run in uncovered containers leading to liquid loss due to evaporation as well as dust deposition. In order to avoid these unintended alterations, recent studies recommend alternative indirect sonicators based on their effective energy delivery as well as suspension purity assurance^6^.

Non-optimized sonication can have a detrimental effect on results. Potentially, it can alter the key physical and chemical properties of the nanomaterials such as size, size distribution, morphology, and surface charge^2^,^9^. Previous literature has reported such failings to control the sonication process and the impact on particle parameters such as nano-TiO_2_^5^,^10^,^11^, nano-ZnO^6^, and nano-copper^12^. Furthermore, past studies have shown that the sonication process not only alters particle characteristics but also governs the outcome of toxicological tests^12^,^13^.

To have control over the dispersion process, it is important to monitor and understand how different factors such as sonicator type, instrument power and duration, volumes, *etc.*, can affect dispersion quality. Hence, there is a need to have a systematic procedure to analyze key physicochemical characteristics of the particles in the dispersion at different time points of the sonication process. Although such considerations have been taken into account by a few researchers, work in this area is limited. Bihari *et al.* have studied dispersion stability of different nanomaterial dispersions made using different ultrasound energies with various dispersion stabilisers^14^. A recent review by Hartmannn *et al.* highlighted that although work has been done to understand the different factors affecting nanomaterial dispersion quality *e.g.*, type of sonicator used, sonication time, *etc.*, there is still no well-defined and universally accepted sonication procedure that currently supports nanotoxicological testing and investigations^7^,^15^.

Several analytical characterization techniques are used to monitor dispersion quality. These include the use of: Dynamic Light Scattering (DLS), Disc Centrifugation, Electrophoretic Light Scattering (ELS), Ultraviolet-visible (UV-vis) spectroscopy, and Transmission Electron Microscopy (TEM), which measure particle size/size distribution, zeta potential, dispersion stability, and morphology characteristics, respectively. DLS is often used to determine the hydrodynamic diameter (Z-average) of the particles and polydispersity index (PdI) of nanomaterial dispersion. In the case of multimodal size distribution by DLS, Z-average obtained may not agree with the intensity-weighted size distribution intensity. As such, the mean of the intensity-weighted size distribution can be quoted. PdI reflects the broadness of the size distribution with a scale ranging from 0 - 1, with 0 being a monodispersed sample and 1 being a highly polydisperse sample^16^. Disc Centrifugation is a separation technique used to determine particle size distribution using centrifugal sedimentation in a liquid medium. The particles sediment within an optically clear and rotating disc and the amount of light scattered by the particles when they reach the edge of the disc is recorded and converted into particle size distribution using Stokes' law. To resolve multi-modal particle distribution, techniques such as disc centrifuge are more suitable as they have a separation mechanism element integrated within the instrument. Zeta potential (*ζ-*potential) of particles is defined as the electric potential at their shear or slipping plane, which is a notional boundary within the electrical double layer that separates the (bulk) liquid showing normal viscous behavior from the Stern layer, a layer that is predominantly composed of counter ions and considered to move with the particle. The zeta potential is directly related to the surface charge of particles and therefore the electrostatic interaction (*i.e.*, repulsion/attraction) between the particles. This parameter is therefore considered a primary indicator of nanomaterial dispersion stability. By convention, zeta potential value below -25 mV and above 25 mV are considered stable^17^,^18^. The concentration and type of ions as well as the solution pH, strongly affect the zeta potential^19^. ELS is used to measure the electrophoretic mobility of particles in dispersion and this mobility is converted to zeta potential through the Henry equation and the Smoluchowski or Hückel models. UV-vis spectroscopy is a technique used to quantify the light that is absorbed and scattered by a sample at a particular wavelength. It is often used to monitor dispersion stability by measuring the characteristic absorption of nanomaterials in the UV region. Finally, TEM is often used to visualize and analyze the size, size distribution, agglomeration, and shape of the nanoparticles^5^,^14^,^15^,^20^.

We present a comparative study of six different nanomaterial dispersions made using ultrasonic bath and an ultrasonic probe fitted with a vial tweeter. The particle concentration, temperature, sonicator type, and settings used in the study are specified in the protocol, so that the experimental settings for similar probes and ultrasonic baths can be inferred. The following nanomaterials are used: silver (Ag), cerium oxide (CeO_2_), zinc oxide (ZnO, NM110-hydrophylic and NM111-hydrophobic), and carbon-based nanomaterials such as carbon nanotubes (A32 and A106, see **Table of Materials**).

Assessment of dispersion quality at different time points along the sonication process is made using various characterization techniques, namely DLS for particle size/size distribution, Disc Centrifugation for size distribution, ELS for zeta potential, UV-vis spectroscopy for stability, and TEM for particle shape and homogeneity. A number of different nanomaterials ranging from metal oxides to carbon-based are evaluated. For comparison, commercial aqueous suspension of silver nanoparticles (Ag NPs) stabilized with citrate capping is used in parallel, to deduce the expected long-term stability of a relevant commercially available suspension. Obviously, this Ag NPs model is not directly related to any of the dispersion procedures but solely acts to indicate the need to re-sonicate or re-stabilize the suspensions after some time of storage as changes such as re-agglomeration are bound to occur during storage. The suspension is kept in the fridge for two months. During this period, the dispersion is characterized to identify potential agglomeration of the particles. Initial results show an unstable suspension (as discussed in the **Results** section). Subsequently, this dispersion is further subjected to different sonication treatments, similar to the other nanomaterials used in the study. The purpose of the study is to confirm that we can de-agglomerate the suspension through the same sonication protocol. The Ag NPs model can thus be associated as the benchmark for long term studies representing re-dispersion of particles in optimized form.

The dispersion protocols presented here share similarities to those published in earlier literature and incorporates some of the few recommendations previously made by past workers^7^,^21^,^22^,^23^,^24^,^25^. In this study, a systematic and step-wise approach is used to monitor dispersion quality throughout the dispersion protocol. This approach undertakes real-time characterization of the nanomaterial dispersions, in order to identify optimal experimental dispersion conditions (**Figure 1**).

**Figure:**
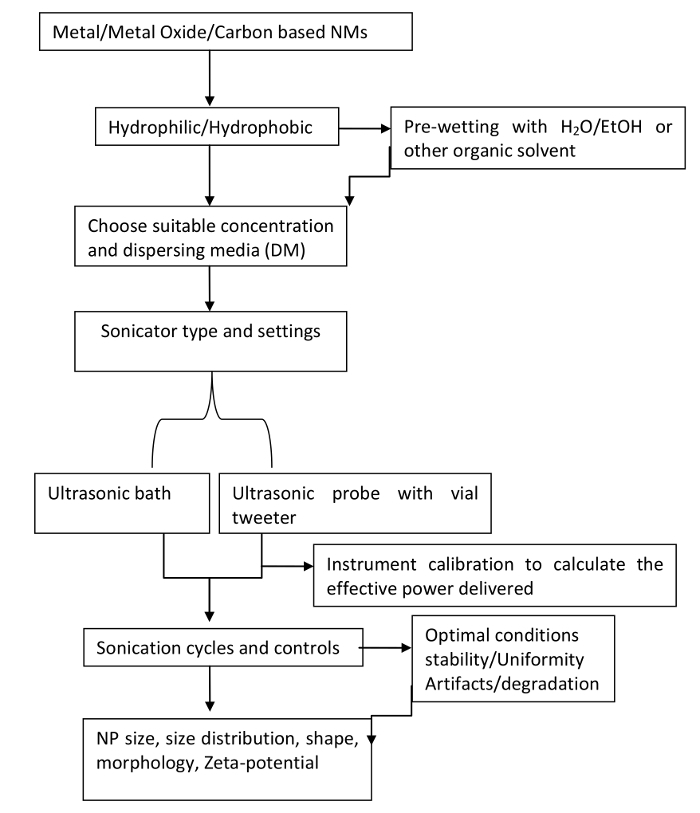

**Figure 1. Flowchart depicting the scheme and step-wise sequence of the dispersion protocol.** Please click here to view a larger version of this figure.

## Protocol

NOTE: All chemicals are used as received without further purification. Use ultrapure water throughout the study with a resistivity of 18 MΩ·cm. All the prepared dispersions are generally stored at 5 °C in the dark for any further characterization or future stability studies but this can vary depending on the material composition and other associated properties like dissolution. For example, Ag NPs are generally stable for some time if stored between 2 - 5 °C away from sunlight; however, dynamic changes are bound to happen within the suspensions and dispersions will re-agglomerate nevertheless and are known to sediment down with time. Analyze these materials using DLS, UV-vis, and TEM for quality verification before biological testing^4^,^5^,^13^,^14^. A concentration of 0.02 mg/mL is used for analysis below. The sample concentration is suitable for analysis using DLS, UV-vis, disc centrifuge, zeta-potential calculations, and TEM analysis.

### 1. Calibration of Delivered Power by Sonicators

NOTE: The effective acoustic power delivered to the sonicated suspension is an important parameter in order to obtain reproducible dispersions. This is different from the electrical input or output power of the generator indicated by the manufacturer as this is the actual power that is deliverable to the suspension during sonication^26^. Amongst many methods for the calculation of effective delivered power, the most commonly used method is calorimetry^26^. This is known to be a simple and efficient way for the direct measurement of effective power delivered to a suspension^7^. In this method, the temperature increase in the liquid at a given sonicator setting is recorded over time and the effective power delivered is calculated using the following equation: 
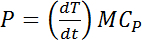
 where P is the delivered acoustic power (W), T is the temperature (K), t corresponds to the time (s), C_P_ is the specific heat of the liquid (4.18 J/g·K for water), and M is the mass of the liquid (g).

1. **Calibration of delivered power by ultrasonic probe fitted with a vial tweeter** NOTE: The method is adapted from Taurozzi *et al.*^7^ and the following steps are recommended.
  1. Place an empty plastic vial on the microbalance and tare the balance.
  2. Fill the vial with 1.5 mL of DI water (resistivity 18 MΩ·cm) and record the mass of the liquid using the balance.
  3. Place the vial in one of the six vial holes in the high intensity part of the vial tweeter and immerse a temperature probe connected to a digital temperature meter using a clamp. Make sure that the probe does not touch the walls of the vial and is approximately 2 cm below the liquid surface.
  4. Set the vial tweeter setting at 24 kHz and 10 W (amplitude adjustment at 50%) and operate in the continuous mode. NOTE: Other amplitude adjustments tested here are 70%, 90%, and 100%.
  5. Record the increase in water temperature for the initial 5 min at an interval of 30 s and ensure that the vial or the set up does not shift position.
  6. Create a temperature versus time graph in a spreadsheet software and obtain the best linear fit for the curve using least squares regression.
  7. Obtain the slope of the graph (which is the rise of temperature over time) and calculate the power delivered using **Equation 1**. Repeat the experiment three times and obtain the mean value.
  8. Repeat the procedure from steps 1.1.1 - 1.1.4 for 70%, 90%, and 100% amplitude settings. The power value obtained using this procedure is reported during dispersion procedure.
2. **Calibration of delivered power by an ultrasonic bath**
  1. Place an empty plastic vial on the microbalance and tare the balance.
  2. Fill the vial with 1.5 mL of DI water (resistivity 18 MΩ·cm) and record the mass of the liquid using the balance.
  3. Place the vial in the middle of the ultrasonic bath half dipped in water and secure it with a clamp. Immerse a temperature probe connected to a digital temperature meter using a clamp. Make sure that the probe does not touch the walls of the vial and is approximately 2 cm below the liquid surface.
  4. Set up the ultrasonic bath at 40 KHz and 80 W and operate in the continuous mode.
  5. Record the increase in water temperature for the initial 5 min at an interval of 30 s and ensure that the vial or the set up does not shift position.
  6. Create a temperature versus time graph in excel and obtain the best linear fit for the curve using least squares regression.
  7. Obtain the slope of the graph in a spreadsheet software (which is the rise of temperature over time) and calculate the power delivered using **Equation 1**. Repeat the experiment three times and obtain the mean value. The power value obtained using this procedure is reported during the dispersion procedure.

### 2. Dispersion Procedure in Aqueous Medium Using an Ultrasonic Probe Fitted with a Vial Tweeter

1. Weigh 2 mg of each of the required nanopowder using a clean spatula into three clean glass vials. Label them as vials 1, 2, and 3.
2. Pipette out 1 mL of DI water and add along the walls of each vial. Make a thick paste with the help of a clean thin glass rod, then add the rest of the water to make a final concentration of 0.2 mg/mL. In case of a hydrophobic sample, perform the pre-wetting using 1 mL of 0.5% vol/vol ethanol and add DI water to make up the required final concentration.
3. Seal each vial with its cap and shake well in the horizontal circular motion to removeany nanopowder sticking to the walls of the vial.
4. Place the three vials in the ultrasonic probe fitted with a vial tweeter and apply the first sonication treatment for 2 min at 1.1 W in pulsed mode (1 s/1 s, which means 1 s on and 1 s off). This will give a temperature rise of about 4 °C in the dispersion.
5. Take out vial 1 and pipette out a suitable amount of aliquot from the top of the vial, dilute it with DI water to a concentration of 0.02 mg/mL. Characterize the diluted dispersion for size, particle size distribution, shape, agglomeration, and zeta potential using a range of complimentary techniques such as DLS, TEM, UV-vis, and ELS (discussed in Section 4). Record and document the measurements accurately.
6. Pause for 10 min from step 2.4 to allow cooling of the sample and to avoid any abrupt temperature rise in the system. Apply a second sonication treatment to vials 2 and 3 for 4 min at the same settings of amplitude and pulsed mode. Take out vial 2, repeat step 2.5, and document the readings after 6 min of sonication.
7. Pause for 10 min, apply a third sonication treatment to vial 3 for another 4 min and then follow step 2.5. Record and document the measurements at 10 min of sonication (discussed in Section 4). NOTE: Lab coats, gloves and goggles must be worn when handling suspensions of nanoparticles. The sonicator must be placed in acoustic enclosure during longer experiments, and high protection ear muffs must be worn when working closer to the ultrasound source.

### 3. Dispersion Procedure in Aqueous Medium Using an Ultrasonic Bath

1. Weigh 2 mg of each of the required nanopowder using a clean spatula in four clean glass vials and label them as vials 4, 5, 6, and 7.
2. Pipette out a few drops of DI water and add along the walls of each vial, and make a thick paste with the help of clean thin glass rod. Then add the rest of the water to make a final concentration of 0.2 mg/mL in each vial. NOTE: In case of a hydrophobic sample, the pre-wetting is carried out using 1 mL of 0.5% vol/vol ethanol and then DI water is added to make up the required final concentration.
3. Seal each vial with its cap and shake well in the horizontal circular motion to remove any nanopowder sticking to the walls of the vial.
4. Place the four vials in the middle of ultrasonic bath with the vials half dipped in water and apply the first sonication treatment at 80 W for 15 min at room temperature. This would give a temperature rise of about 3 °C in the dispersion.
5. Remove vial 4 from the ultrasonic bath and pipette out a suitable amount of aliquot from the top of the vial, dilute it with DI water to a concentration of 0.02 mg/mL, and characterize the sample for size, particle size distribution, shape, agglomeration, and zeta potential using a range of complimentary techniques such as DLS, TEM, UV-vis, and ELS (discussed in Section 4). Record and document the measurements.
6. Change the water in the ultrasonic bath and apply a second sonication treatment to vials 5, 6, and 7 for another 15 min at the same settings (80 W). Remove vial 5, follow step 3.5 for characterization and document the readings at 30 min of sonication.
7. Change the water in the ultrasonic bath (to avoid any further temperature rise) and apply a third sonication treatment of another 30 min to vials 6 and 7 at the same settings with a small pause of change of water again at 15 min. Remove vial 6 and follow step 3.5. Record and document the measurements at 1 h of sonication.
8. Change the water again in the ultrasonic bath every 15 min and apply a fourth sonication treatment to vial 7 for another hour keeping the settings constant. Take out vial 7 and follow step 3.5 for complete characterization, and record the measurements at 2 h of sonication.

### 4. Characterization of the Dispersed Samples at Different Time Points

1. **Size characterization using DLS** 27
  1. Open the DLS software. Create a size measurement file that can be individualized for a specific nanomaterial (including one for a standard) using the refractive index value from the Malvern manual. In addition, input any other data as required by the software, such as values of absorption and viscosity, and also type of dispersant.
  2. Enter in the experiment conditions for the sample, such as 2 min equilibration time, 20 °C temperature, cuvette type as low volume disposable cuvette, and experiment running in automatic mode. Press File|Save (save with the desired name).
  3. Press "File|Open new measurement" and run a DLS verification test by using standard latex beads with a nominal size of 100 nm to qualify instrument performance
    1. Use a low volume disposable cuvette. Inject 1 mL of sample using syringe or pipettes to avoid any air bubbles. NOTE: Clean the cuvettes with ethanol and DI water before use.
    2. Insert the cuvette into the machine. Click on the "start" button in the file measurement panel. Note that this will equilibrate the sample for 2 min and take measurements at 20 °C. NOTE: If samples have been previously stored in the fridge, allow them to reach room temperature prior to use.
    3. Collect at least five measurements in automatic mode and take the average of the measurements to report the size by selecting all the measurements and clicking "average" from the top panel. Export the data to excel for further analysis.
    4. Report the hydrodynamic diameter as the Z-average, with the PdI width representing the standard deviation of the Z-average in case of a monomodal distribution^28^. In case of a significant discrepancy between the Z-average and the mean of the intensity-weighted size distribution, which is indicative of polydispersity or agglomeration, the mean of the intensity-weighted size distribution result is quoted with a comment on the sample status.
    5. Repeat step 4.1.3 for new measurements. NOTE: DLS is not a suitable technique for analysis of non-ideal samples. By this we mean samples which are non-spherical particles of high polydispersity, extensive agglomeration, sedimentation, *etc.* The repeated measurements may result in inaccurate readings due to sedimentation/settling particles. In such cases, other complimentary techniques are recommended such as disc centrifugation, which can be used to assess the dispersion in a qualitative manner.
2. **Size distribution by Disc Centrifugation**
  1. Open the CPS software. Select "Procedure definition", put in the sample SOP name at the top, and fill the sample parameters such as minimum and maximum diameter, particle density, refractive index, absorption, and non-sphericity factor^29^.
    1. For example, for ZnO nanoparticles, enter 0.1 micron and 1.0 micron in the minimum and maximum diameter tabs, respectively, enter 5.61 g/mL in the particle density, 2.1 in the refractive index section, 0.001 in the particle absorption, and 1 in the non-sphericity section.
  2. Fill the calibration standard details based on the PVC standard of peak diameter 0.377 µm with a particle density of 1.385 g/mL. Also fill in the fluid parameters (sucrose, fluid density of 1.04 g/mL, and fluid refractive index of 1.35), and name and save the procedure.
  3. Chose the selected procedure (SOP saved in step 4.2.1) and inject the first gradient level, 1.6 mL of sucrose (24%) into the hole through the disc and press 'start'. NOTE: The role of sucrose here is to establish a density gradient within the disc while spinning at a constant speed. This automatically calculated the disc velocity depending on the size range.
  4. Wait until the software reaches the automatically calculated RPM (rotations per minute). Stabilize the sedimentation by injecting a gradient of sucrose (8% low density and 24% high density, see **Table 1**), 1.6 mL total volume each time starting with highest density and ending with the lowest density solution. NOTE: Here we mark the 8% sucrose solution as low and 24% sucrose solution as high. They are mixed in following volumes (total volume 1.6 mL each time) and injected into the disc one by one until a gradient is formed.
    1. Following this, inject 1.0 mL of dodecane cap fluid which helps maintain the gradient inside the disc for a minimum of 6 h. Allow the disc centrifuge to equilibrate for 1 h.
  5. Select "Operate analyzer" and introduce the sample ID, and press start. Inject 0.2 mL of standard with a 1 mL syringe in the disc and press the space bar at the same time. Then inject 0.2 mL of the sample and press the space bar at the same time. Wait for the measurement to finish and then click on next sample.
  6. Use the disc centrifuge control system software to acquire and process the data. For this, click "retrieve distribution" and click on the sample name; this opens the size distribution graph for the sample. Export the data to a spreadsheet manager.
3. **Dispersion stability study using UV-vis spectroscopy** NOTE: UV-vis spectroscopy is often used to understand the suspension stability and aggregation by carefully observing the changes in the peak intensity, spectral skewness, spectral shape as well the wavelength shift in the absorption spectrum^4^. The detailed steps are as follows.
  1. Open the UV-vis spectrophotometer software and click "spectrum scan"^30^.
  2. Use a standard quartz cuvette (semi microrectangular quartz cell 100 mm, 190 - 2,700 nm). Inject 2 - 3 mL of sample using a pipette.
    1. Before use, wash the cuvettes with 50% nitric acid for 10 min and then wash three times with purified water. Then rinse with acetone, remove excess, and air dry.
  3. Preset the instrument setting range to 700 nm to 200 nm wavelength from the wavelength tab by clicking "Instrument" at the top of the software panel and clicking 'set wavelength'.
  4. Click "baseline". Background subtract each spectrum using a corresponding 'blank' *i.e.*, a cuvette filled with only dispersing medium, which in this case is water. NOTE: In case of hydrophobic samples, a similar ratio of ethanol:water is used as the dispersing medium.
  5. Collect at least three individual spectra on each sample by clicking "Instrument| Property", and enter '3' in the number of spectra. Take average values for analysis. Save the data and export the data for further analysis.
4. **Zeta potential measurements using ELS**
  1. Open the DLS software. Create a zeta potential measurement file that can be individualized for a specific nanomaterial using the refractive index value from the Malvern manual. Input other information that can be plugged into the software *e.g.*, absorption, viscosity, and type of dispersant as found in the sample setting tab. Click "File|Save" and save with the desired name.
  2. Click "File|Open new measurement" and verify the instrument performance by using a reference standard DTS 1235 (zeta potential standard). This is a polystyrene latex standard in aqueous buffer at pH 9 and has a zeta potential of -42 ± 4.2 mV.
  3. Prepare the sample in a syringe of at least 1 mL capacity. Use a disposable folded capillary cell fitted with an electrode on each side for the zeta potential measurements. Carefully inject the sample into the capillary cell through one of the ports on the capillary cell checking that there are no bubbles.
    1. Once the sample starts to emerge from the other end, insert the stoppers and remove any liquid which may have spilt onto the electrodes. Clean the cuvettes thoroughly with ethanol and DI water.
  4. Insert the folded capillary cell into the machine. Equilibrate for 2 min and acquire the measurements at 200 °C unless specified. If dispersion samples have been previously stored in the fridge, allow the dispersion samples to reach room temperature prior to use.
  5. Collect at least five measurements in automatic mode and report the averaged zeta potential value. Export the data, analyze^17^,^18^ (typically, zeta potential value below -25 mV and above 25 mV is considered stable), and interpret online or offline.
5. **Morphological characterization using TEM**
  1. Use grids (300 mesh) holey carbon films for the sample preparation. Put a drop of the dispersion sample (approximately 0.1 mL, 0.02 mg/mL) on a clean grid.
  2. Allow sample to air dry in ambient conditions whilst keeping the grids covered to prevent airborne contamination.
  3. Wash the grids with ultra-pure water to remove any drying effects, and subject to TEM imaging. NOTE: The addition of the dispersion drop on the grid increases the concentration of particles on the grid surface thus leading to attractive inter-particle forces. Uneven drying can lead to artifacts. A small rinse with ultra-pure water eliminates this risk and is helpful for a uniform drying of the grids^31^.
  4. Acquire the images in a dm3 format and later examine them offline using TEM software. NOTE: Images can be used to deduce complementary information surrounding particle size, structure and shape. Files are converted to tiff, in which quantification on properties such as shape and size can be carried out.

## Representative Results

The calorimetric data showing the rise in temperature over time during both sonication types are shown in Figure 2. The effective acoustic power delivered to the dispersion in an ultrasonic probe fitted with a vial tweeter (power source 200 W) is calculated to be 0.55 ± 0.05 W at 50% amplitude, 0.75 ± 0.04 W at 70% amplitude, 1.09 ± 0.05 W at 90% amplitude, and 1.15 ± 0.05 W at 50% amplitude, whereas for the ultrasonic bath (power source 80 W), it is calculated to be 0.093 ± 0.04 W at 100% setting. The finding is similar to previously published work, which demonstrates that the power output displayed by the sonicators is far less than that delivered to the suspensions under treatment^32^,^33^,^34^.

**Figure:**
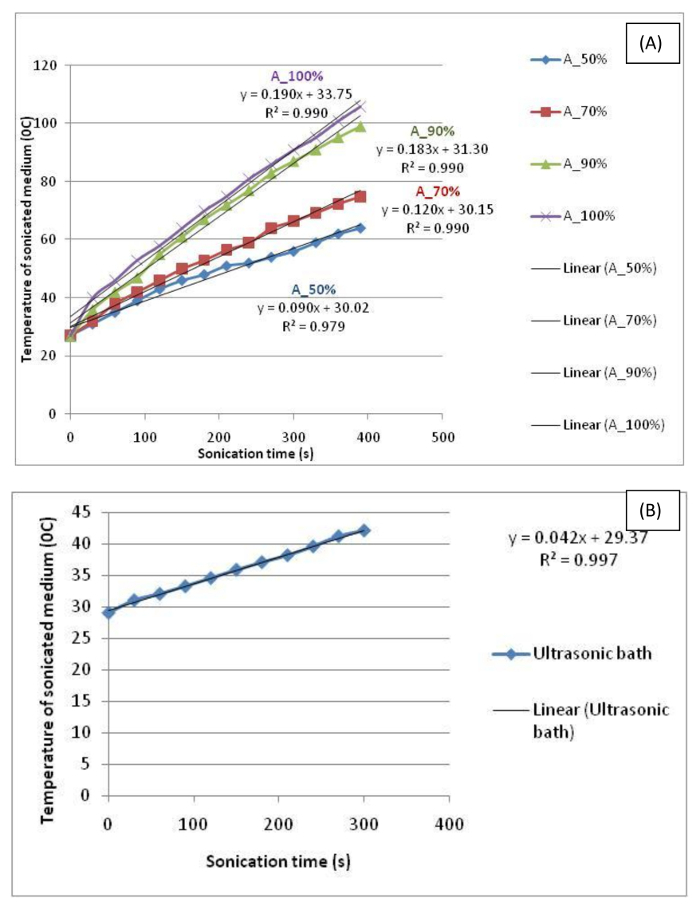

**Figure 2. Calorimetric data showing temperature increase over time during sonication using (A) an ultrasonic probe fitted with a vial tweeter and (B) an ultrasonic bath.** The effective acoustic power delivered to the dispersion in an ultrasonic probe fitted with a vial tweeter (power source 200 W) is calculated to be 0.55 ± 0.05 W at 50% amplitude, 0.75 ± 0.04 W at 70% amplitude, 1.09 ± 0.05 W at 90% amplitude, and 1.15 ± 0.05 W at 50% amplitude, whereas for the ultrasonic bath (power source 80 W), it is calculated to be 0.093 ± 0.04 W at 100% setting. Please click here to view a larger version of this figure.

Findings associated with the various nanomaterials dispersions produced by different protocols are summarized in **Table 2**. Results show the variability in the dispersion quality (as measured by DLS, ELS, and TEM) associated with different nanomaterials dispersions produced using different sonication conditions. As expected, data variability is governed by several factors such as type of nanomaterial, sonication time period, and whether a probe or an ultrasonic bath has been used in the protocol. The UV-vis spectrum obtained for each nanomaterial is shown in Figure 3 and Figure 4 and the DLS results are shown in Figure 5 and Figure 6.

The purpose of **Table 2** is not only to show the degree of data variability but also to allow identification of an optimized dispersion protocol for a given nanomaterial dispersion. If such dispersions had been used as part of a nanotoxicological test method, then the ideal is to have a stable dispersion (preferably a magnitude of at least ± 30 mV), a small PdI indicating narrower particle size distribution (preferably with PdI of 0.2 or less), and a small mean DLS particle size, to indicate the breakup of large agglomerates. Here, Z-average is defined as the intensity based average size of the nanoparticles and PdI is a measure of the width of the overall size distribution (described above in the **Introduction**).

**Table:** 

**NM**	**Sample code**	**Sonication time**	**Size by DLS (nm)**	**Polydispersity Index (PdI)**	**Zeta potential (mV)**
**Cerium oxide**	CeO2_powder	0	396±130	0.763±0.100	17.2±0.4
CeO2_B_15min	15 min	128±4	0.231±0.015	39.2±1.0
CeO2_B_30min	30 min	117±5	0.210±0.008	38.1±0.5
CeO2_B_1h	1 h	95±3	0.209±0.012	46.5±0.5
CeO2_B_2h	2 h	92±2	0.203±0.007	46.5±1.4
CeO2_P_2min	2 min	126±7	0.218±0.005	28.8±0.7
CeO2_P_6min	6 min	131±2	0.209±0.014	40.5±0.7
CeO2_P_10min	10 min	122±1	0.184±0.014	44.4±1.3
**Zinc Oxide (hydrophilic)**	ZnO_NM110 powder	0	1410±120	0.786±0.150	17.1±0.5
ZnO_NM110_B	15 min	239±2	0.130±0.024	25.4±1.0
_15min
ZnO_NM110_B	30 min	251±2	0.166±0.020	21.6±0.3
_30min
ZnO_NM110_B	1 h	310±8	0.162±0.025	21.0±0.2
_1hr
ZnO_NM110_B	2 h	274±3	0.243±0.014	25.2±0.7
_2hr
ZnO_NM110_P	2 min	377±20	0.267±0.025	21.7±0.4
_2min
ZnO_NM110_P	6 min	885±70	0.276±0.023	8.6±0.6
_6min
ZnO_NM110_P	10 min	1074±88	0.673±0.058	11.2±1.4
_10min
**Zinc Oxide (hydrophobic)**	ZnO_NM111_	0	758±86	0.823±0.006	-14.6±0.7
powder
ZnO_NM111_	15 min	384±95	0.399±0.074	-17.5±1.0
B_15min
ZnO_NM111_	30 min	282±35	0.361±0.009	-22.4±0.5
B_30min
ZnO_NM111_	1 h	296±18	0.379±0.031	-22.8±0.5
B_1hr
ZnO_NM111_	2 h	280±54	0.366±0.031	-23.7±1.0
B_2hr
ZnO_NM111_	2 min	227±9	0.402±0.032	19.8±0.8
P_2min
ZnO_NM111_	6 min	340±58	0.477±0.026	-21.1±0.2
P_6min
ZnO_NM111_	10 min	370±72	0.626±0.065	-21.8±0.8
P_10min
**CNT**	A32_powder	2 min	306±5	0.279±0.029	-23.7±0.5
A32_B_15min	15 min	250±3	0.200±0.007	-18.0±0.4
A32_B_30min	30 min	255±2	0.282±0.036	-20.2±1.1
A32_B_1hr	1 h	230±3	0.226±0.021	-21.7±0.5
A32_B_2hr	2 h	267±3	0.337±0.019	-20.6±0.6
A32_P_2min	2 min	255±4	0.217±0.011	-22.5±0.4
A32_P_6min	6 min	245±9	0.328±0.029	-23.6±0.8
A32_P_10min	10 min	254±4	0.313±0.029	-23.6±0.5
**CNT**	A106_powder	2 min	580±18	0.305±0.070	-35.9±1.0
A106_B_15min	15 min	573±18	0.404±0.016	-29.5±1.0
A106_B_30min	30 min	479±11	0.363±0.013	-28.8±1.4
A106_B_1hr	1 h	566±22	0.461±0.054	-25.0±0.7
A106_B_2hr	2 h	477±10	0.311±0.027	-26.8±0.5
A106_P_2min	2 min	300±58	0.473±0.053	-29.8±1.0
A106_P_6min	6 min	390±10	0.359±0.022	-40.7±0.5
A106_P_10min	10 min	300±85	0.511±0.134	-24.5±0.7
**Silver**	Ag_cit	0	72±50	0.462±0.258	-38.7±1.3
Ag_B_15min	15 min	25±1	0.489±0.008	-39.8±2.2
Ag_B_30min	30 min	25±1	0.532±0.036	-30.7±2.8
Ag_B_1hr	1 h	25±1	0.542±0.028	-39.2±1.7
Ag_B_2hr	2 h	28±5	0.387±0.015	-35.8±1.8
Ag_P_2min	2 min	29±1	0.300±0.025	-42.0±2.9
Ag_P_6min	6 min	26±2	0.263±0.017	-40.4±1.5
Ag_P_10min	10 min	25±2	0.251±0.011	-47.3±1.4

**Table 2. Summary of the results of NM dispersion in water. **'P' in the sample codes indicate dispersion carried out using an Ultrasonic probe fitted with a vial tweeter and 'B' in the sample code indicated dispersion carried out using an ultrasonic bath. All the measurements were taken at 0.02 mg/mL. Sonication at time 0 means a non-sonicated suspension *i.e.*, just firm shaking and mixing without any other aid. CNTs that are completely insoluble and non-dispersible in DI water on physical shaking were sonicated for an initial 2 min in the bath sonicator and also reported.

**Figure:**
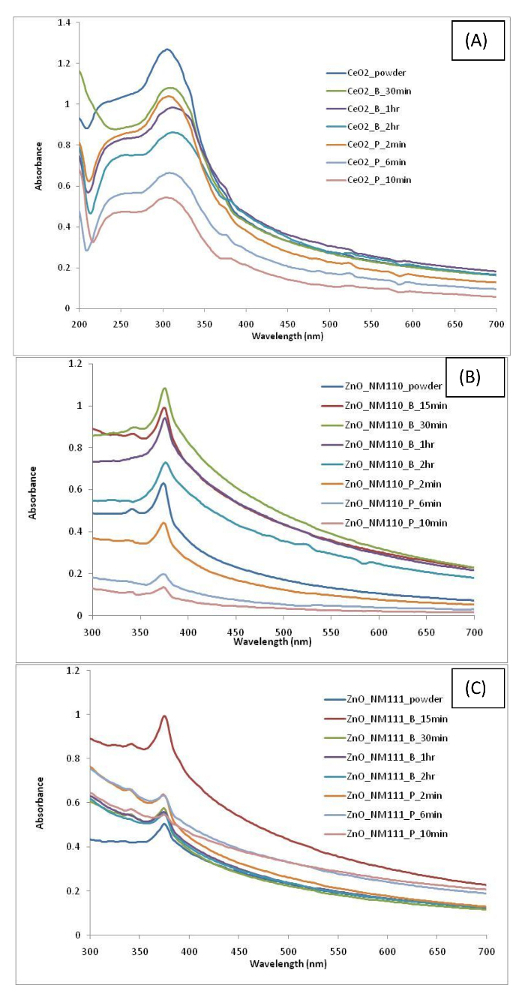

**Figure 3. UV-vis spectra of (A) CeO_2_, (B) ZnO NM110, and (C) ZnO NM111 dispersion in water. **UV-vis spectroscopy is used to understand the suspension stability and aggregation by carefully observing the changes in the peak intensity, spectral skewness, spectral shape as well the wavelength shift in the absorption spectra. Please click here to view a larger version of this figure.

**Figure:**
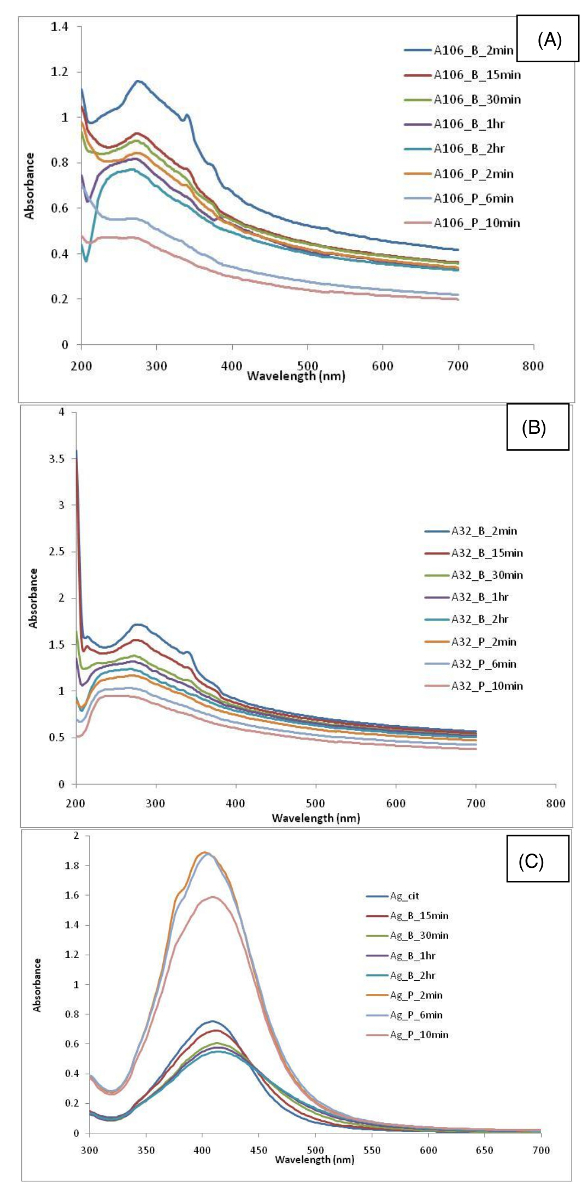

**Figure 4. UV-vis spectra of (A) CNTs A106, (B) CNTs A32, and (C) Ag_citrate dispersion in water.** UV-vis spectroscopy is used to understand the suspension stability and aggregation by carefully observing the changes in the peak intensity, spectral skewness, spectral shape as well the wavelength shift in the absorption spectra. Please click here to view a larger version of this figure.

**Figure:**
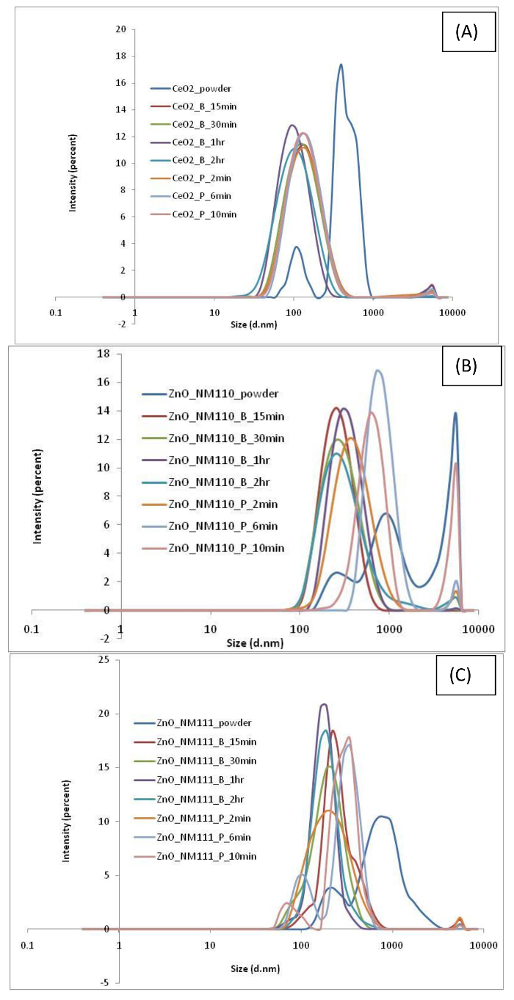

**Figure 5. Size distribution by intensity obtained with DLS for (A) CeO_2_, (B) ZnO NM110, and (C) ZnO NM111 dispersion in water.** Please click here to view a larger version of this figure.

**Figure:**
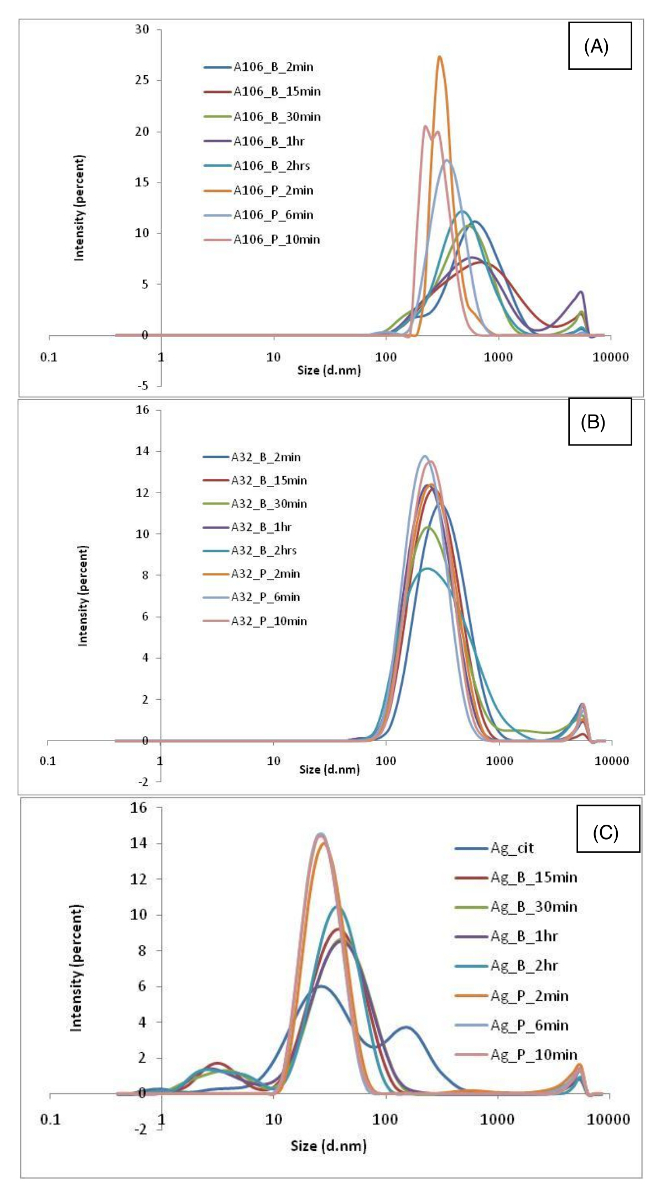

**Figure 6. Size distribution by intensity obtained with DLS for (A) CNTs A106, (B) CNTs A32, and (C) Ag_citrate dispersion in water.** Please click here to view a larger version of this figure.

In the case of CeO_2_ nanomaterial suspension, the use of sonication resulted in an overall decrease in particle size and PDI values. Without any sonication, results show a multimodal intensity distribution with a Z-average (396 ± 130 nm) and a very high PdI value of 0.763 ± 0.100 (**Table 2**). Furthermore, the dispersion shows a zeta potential value of 17.2 ± 0.4 mV. It should be noted that a PdI of ≥0.5 is indicative of a highly polydisperse suspension. Therefore, the sample was subjected to Disc Centrifugation, and the size distribution data obtained also confirmed a non-uniform and inhomogeneous sample (Figure 7a). Sample morphology and size analysis by TEM further confirmed that the particles in the dispersion are indeed highly polydisperse (Figure 8). Upon dispersing the powder using an ultrasonic bath for 15 min, results showed improvement in the overall dispersion quality. In particular, the overall stability (as noted by its corresponding zeta potential value) and monodispersity had improved. Increasing the sonication time to 2 h resulted in much improved stability and narrower particle size distribution (**Table 2**). It is clear that there is gradual improvement in dispersion quality if longer bath sonication time is used, as seen by the gradual decrease in the hydrodynamic diameter and PdI. Similar results were obtained if the dispersion procedure had been carried out using an ultrasonic probe instead. Overall, a more stable and homogenous state of agglomeration has been achieved using the probe, as confirmed by DLS and TEM data. Interestingly, ultrasonic bath proved to be a better option than the use of a probe, as a much smaller mean particle size and a much higher zeta potential value can be achieved using a bath rather than a probe. It is observed that in both sonication procedures, the TEM micrographs confirmed the presence of different primary particles to include: spheres, cubes, and polyhedrons.

**Figure:**
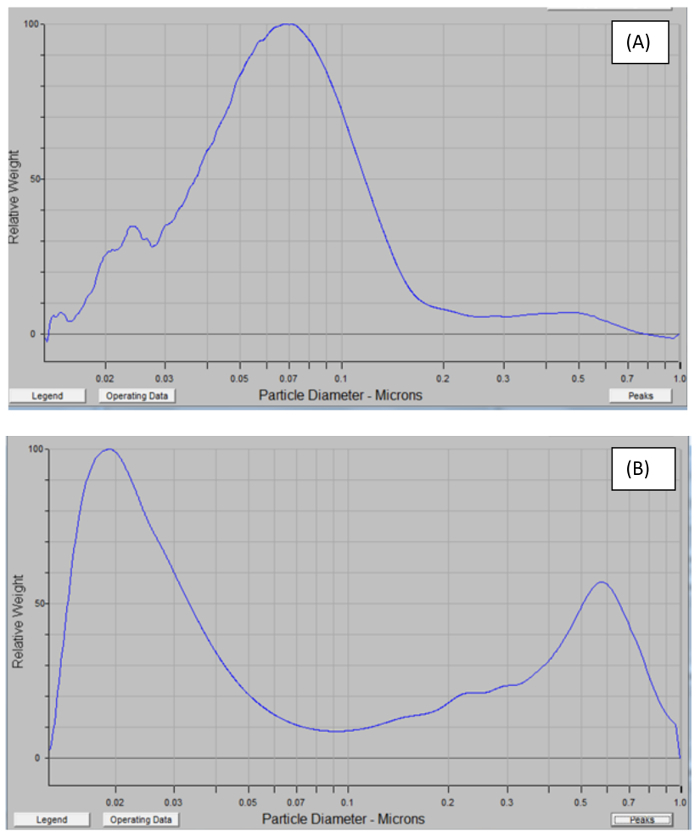

**Figure 7. Size distribution obtained with Disc Centrifugation for (A) CeO_2__powder and (B) ZnO NM110_powder dispersion in water at 0 min.** Please click here to view a larger version of this figure.

**Figure:**
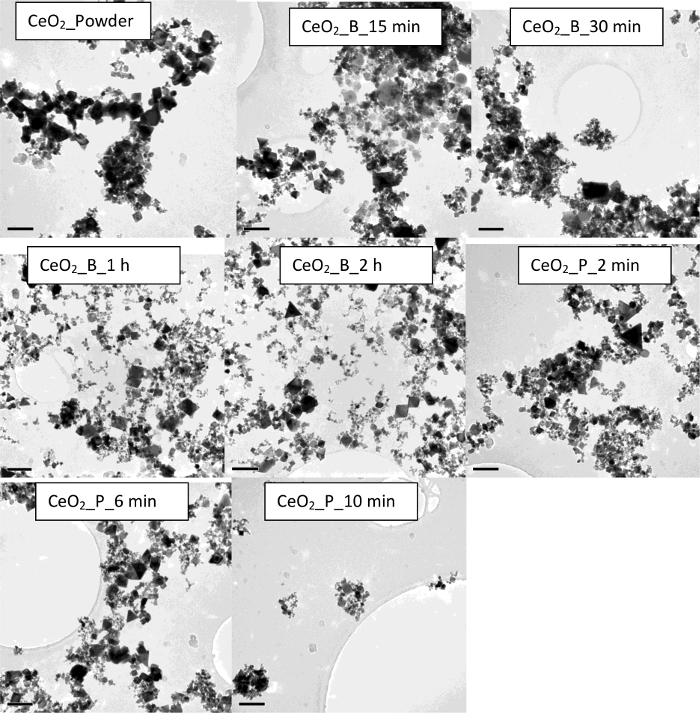

**Figure 8. TEM images of the CeO_2_ demonstrating the impact of sonication on the sample homogeniety and stability. **The scale bar is 100 nm for each sample. Please click here to view a larger version of this figure.

In the case of ZnO, two types of ZnO are used in the dispersions *i.e.*, ZnO nanomaterials of different surface profiles, hydrophilic (NM110) and hydrophobic (NM111). Results indicate similar findings between the two types of ZnO. Both show that with no sonication, the dispersion quality indicated a large particle mean size and high polydispersity. NM110 has a Z-average of 1,410 ± 120 nm and PdI of 0.786 ± 0.150 nm; NM111 has a Z-average of 758 ± 86 nm and PdI of 0.823 ± 0.006. Size distribution data obtained for NM110 from Disc Centrifugation also confirm sample polydispersity and inhomogeneity (Figure 7b). The size and polydispersity of the sonicated NM110 appear to decrease with 15 min treatment in ultrasonic bath and reach an optimal reduction plateau at 30 min sonication time. A longer sonication time shows a general increase in particle size data, potentially due to particle re-agglomerating after being de-agglomerated initially. On the other hand, NM110 shows a homogenous and stable dispersion after 2 min of ultrasonic probe treatment. However, longer cycles of 6 min and 10 min also show an increase in particle size and PdI values, indicating re-agglomeration of the particles. TEM (Figure 9 and Figure 10) and UV-vis (Figure 3b**-c**) results further confirm the state of such dispersion quality. Interestingly, very similar results are observed in the case of NM111 when treated with an ultrasonic probe. Again, the systematic approach indicates that the best dispersion was achieved at 2 min, as possible re-agglomeration may be associated with corresponding 6 min and 10 min cases. When an ultrasonic bath was used instead, the dispersion particle size reached a plateau after 30 min of sonication; after that no further increase or decrease in size or polydispersity values is observed. Also, TEM micrographs obtained for the hydrophobic NM111 indicate the presence of various artifacts and other drying effects on the TEM grid (Figure 10). This shows that pre-wetting with ethanol or other organic solvents may be helpful towards the preparation of aqueous dispersions but there were challenges upon immobilizing hydrophobic nanomaterial samples on the carbon grids. Overall, if an optimal dispersion protocol is identified and if this is governed by the smallest corresponding PDI value, then this corresponds to ZnO_NM110_B1 h and ZnO_Nm111_B30 min for the hydrophilic NM110 and hydrophobic NM 111 cases, respectively.

**Figure:**
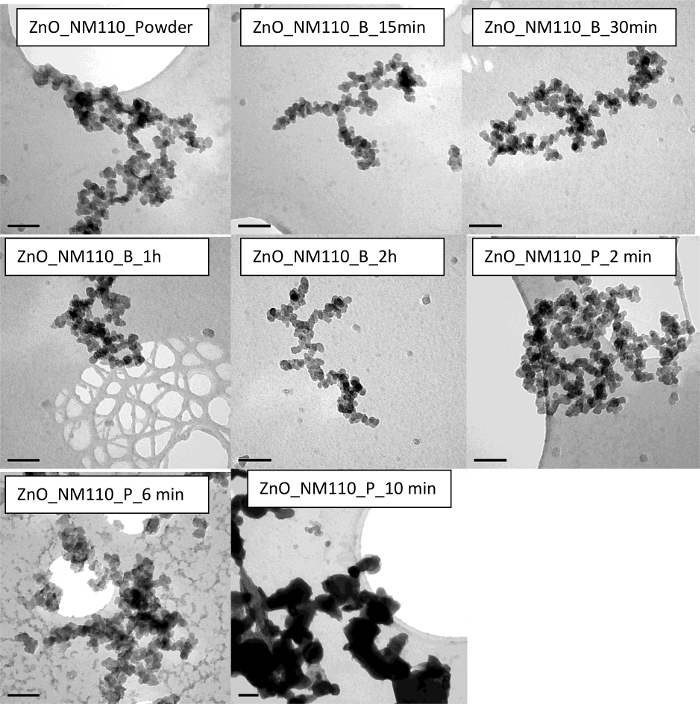

**Figure 9. TEM images of the ZnO NM110 demonstrating the impact of sonication on the sample homogeniety and stability.** The scale bar is 100 nm for each sample. Please click here to view a larger version of this figure.

**Figure:**
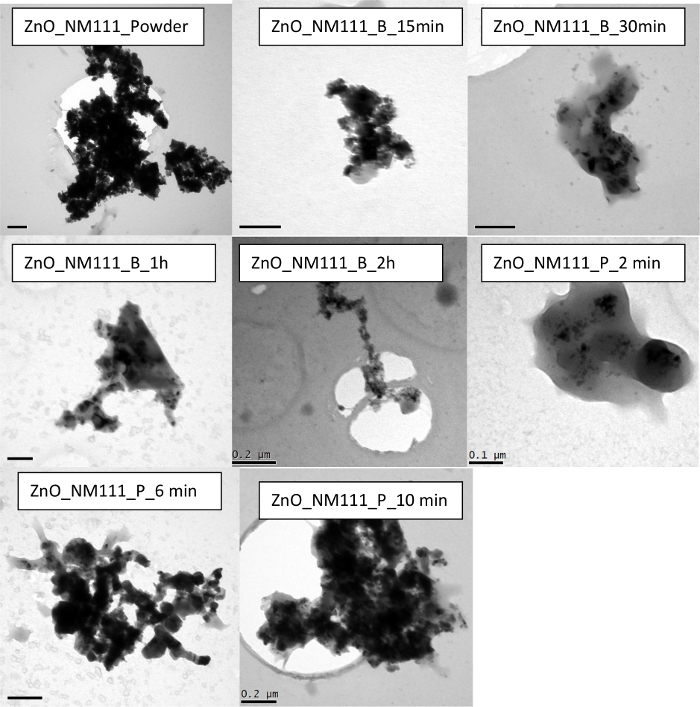

**Figure 10. TEM images of the ZnO NM111 demonstrating the impact of sonication on the sample homogeniety and stability.** The scale bar is 0.1 µm for ZnO_NM111_B_15 min, ZnO_NM111_B_1 h, and ZnO_NM111_P_2 min, and 0.2 µm for rest of the samples**.** Please click here to view a larger version of this figure.

In the case of the carbon nanotubes (CNTs), results show that such nanomaterials are not easily dispersible in water, in particular the dispersion protocol involves the use of physical stirring or vigorous shaking. This is true for both multi-walled carbon nanotubes (MWCNTs) used in this study. TEM micrographs in the case for both A106 and A32 dispersions carried out at 2 min and 15 min of sonication cycle are shown in Figure 11 and Figure 12, respectively. Upon increasing sonication time, results indicate breakage of CNTs, often resulting in length modifications. Such length modifications were apparent in the case of both probe and ultrasonic sonication. Results show that the A106 and A32 CNTs can be sufficiently dispersed after a 2 min treatment if an ultrasonic probe is used. Here sufficient dispersion means the critical sonication time threshold where all the carbon nanotube (CNT) bundles are open and individual tubes are separated^35^. Upon increasing the sonication time to 6 min or 10 min, results indicate a modification of length distribution and much higher polydispersity. Finally, the intensity distributed size data from DLS (Figure 6a**-b**) and the absorption spectra through UV-vis (Figure 4a**-b**) also confirm that CNT dispersions are very sensitive to sonication time and whether a probe or a bath has been used. Both A106 and A32 CNTs show an absorbance peak between 253 and 310 nm, which is typical of MWCNTs^36^. Peak intensity is known to be a good indicator of maximum achievable dispersion in a sonication-driven dispersion of MWCNTs. The UV-spectrum of both A106 and A32 indicates 2 min and 15 min of sonication cycle to be optimum for the suspension. Upon prolonged sonication, the peak broadens with lesser peak intensity as well as sample destruction indicated by the shift in the absorbance spectrum and spectral skewness (formation of peak shoulders).

**Figure:**
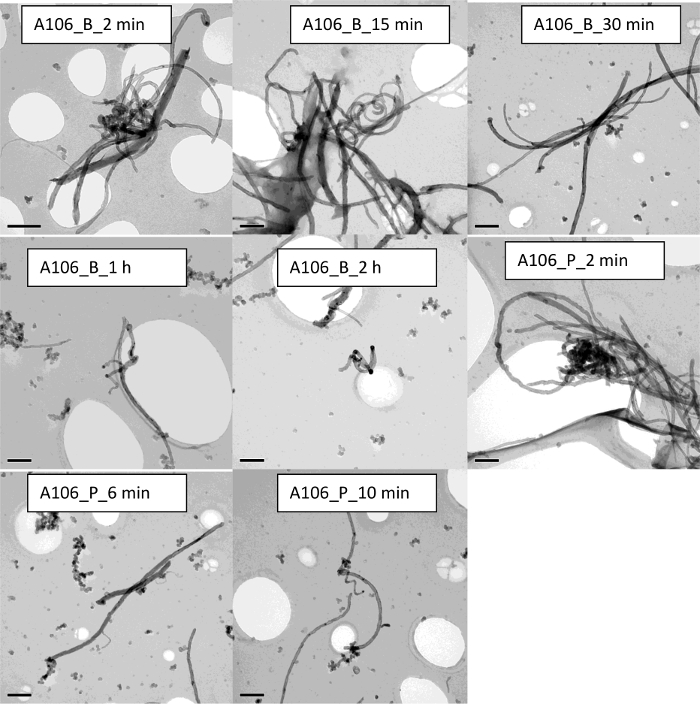

**Figure 11. TEM images of the CNTs A106 demonstrating the impact of sonication on the sample homogeniety and stability.** The scale bar is 200 nm for each sample. Please click here to view a larger version of this figure.

**Figure:**
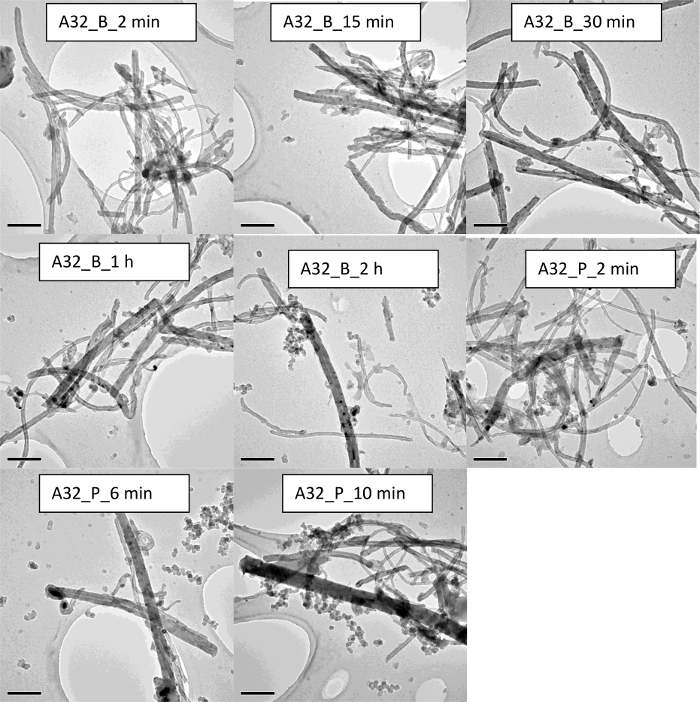

**Figure 12. TEM images of the CNTs A32 demonstrating the impact of sonication on the sample homogeniety and stability.** The scale bar is 200 nm for each sample. Please click here to view a larger version of this figure.

Finally, to have some degree of comparisons, the data are compared to a commercially available suspension of citrate stabilized Ag NPs (nominal diameter of 10 nm, 0.02 mg/mL). Characterization data show that the dispersion is highly agglomerated and highly polydisperse. DLS data show a multimodal distribution with a hydrodynamic diameter of 72 ± 50 nm and a high PdI of 0.46 ± 0.26 (Figure 6c). Morphological analysis by TEM (Figure 13) and wide Surface Plasmon Resonance (SPR) peak (absorption at 418 nm in visible region) by UV-vis (Figure 4c) further confirm a highly polydisperse sample. Interestingly, the ultrasonic bath treatment improves the dispersion stability and PdI, but only if a sufficiently long sonication time period is used; a 2 h sonication time is needed to result in DLS particle size of 28 ± 5 nm and PdI 0.387 ± 0.015 (**Table 1**). However, if an ultrasonic probe is used instead, the sample homogeneity and stability remarkably improve at just 2 min sonication time, thus resulting in DLS particle size of 29 ± 1 nm, PdI of 0.300 ± 0.025, and ZP -42 ± 3 mV. This improvement in dispersion quality is also evident up to a 10 min sonication time setting, in which a DLS particle size of 25 ± 2 nm, PdI 0.251 ± 0.011, and ZP -47.3 ± 1.4 mV is observed. Here, at 10 min of sonication using vial tweeter, the PdI decreases and the ZP increases. The corresponding TEM micrographs at such respective time points also confirm improved sample homogeneity after the appropriate sonication protocols are applied. There is a rapid improvement in the sample homogeneity and dispersity of particles in TEM images. The sample at 2 min shows some agglomeration as compared to the individual particles sonicated for 10 min using the vial tweeter.

**Figure:**
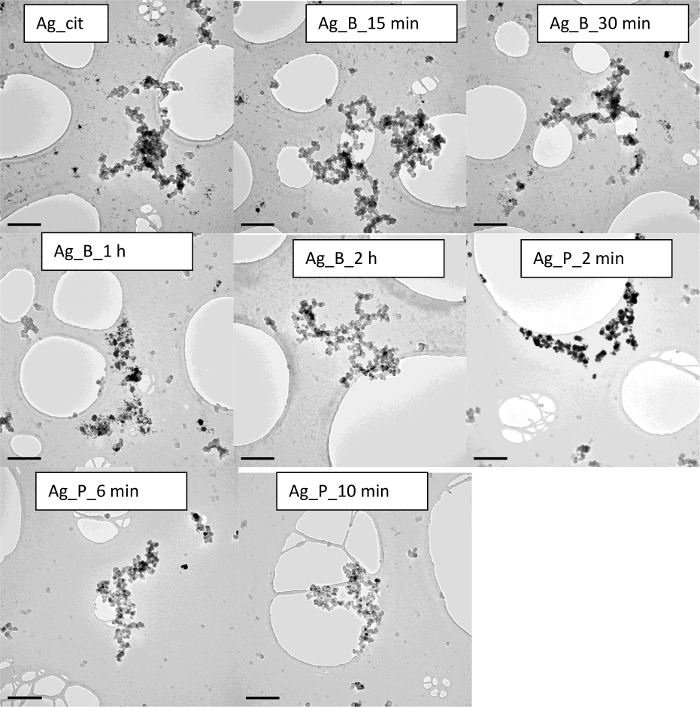

**Figure 13. TEM images of the commercial Ag NPs demonstrating the impact of sonication on the sample homogeniety and stability.** The scale bar is 200 nm for each sample. Please click here to view a larger version of this figure.

**Table:** 

**High (mL)**	**Low (mL)**
1.4	0.2
1.2	0.4
1	0.6
0.8	0.8
0.6	1
0.4	1.2
0.2	1.4
0	1.6

**Table 1. Sucrose density gradient mixing for total 1.6 mL volume. **Here we mark the 8% sucrose solution as low and 24% sucrose solution as high. They are mixed in the following volumes (total volume 1.6 mL each time) and injected into the dis cone one by one until a gradient is formed.

## Discussion

The ultimate goal of the study is to develop a strategy that would allow the identification of optimal sonication conditions to make dispersions of a selected number of nanomaterials in water. An attempt is made here to carefully document the protocol steps and parameters during sonication in order to fulfill the gaps previously identified in reviews as well as to follow the recommendations made in the past^15^. The optimal dispersion conditions are identified by characterizing the dispersions after each sonication cycle and checking sample stability and uniformity. The impact of the sonication procedures and stability status is assessed based on the characteristic alterations in key physicochemical properties of nanomaterials, as determined by various analytical techniques: DLS, ELS, UV-vis, and TEM. The current protocol is an adapted methodology for the dispersion of nanomaterials from the past literature and other research projects^21^,^22^,^37^,^38^,^39^ with some modifications and refinements addressing the key gaps, steps, and their applicability to wider nanomaterials of similar surface profile^7^. However, careful adjustments are required with respect to their sonication time, strength, and type for their application to other nanomaterials. Also, further work is required to establish a correlation between sonication procedure and biological activity of nanomaterials. Six different types of nanomaterial dispersions are evaluated and compared, primarily for their stability, using an ultrasonic bath and an ultrasonic probe fitted with a vial tweeter at set time points. To maintain the suspension purity and any unintended alterations caused due to contamination, probe sonication is avoided here. In the vial tweeter, the vials can remain closed. This eliminates any cross contamination of the samples.

Calibration of sonicators is a key factor since a range of sonicators are available with different frequencies, amplitude, and powers. To determine the effective acoustic energy delivered to the suspension, calibration of sonicators is carried out using calorimetry. The acoustic power delivered for 70% amplitude setting for the vial tweeter as well as that for the 100% ultrasonic bath setting is calculated to be <1 W (0.75 ± 0.04 W and 0.093 ± 0.04 W, respectively). However, the power outputs indicated by the manufacturers for vial tweeter and bath sonicator are 200 W and 80 W, respectively. This indicates that despite the high power source, most of the energy is lost during the generation of cavitational bubbles and only a small fraction is actually delivered to the dispersion under treatment^26^. Recent studies have highlighted the importance of cavitational measurement control as compared to the input power of the sonicator for a better dispersion control during sonication^8^. The methodology appears promising for the controlled dispersion of highly delicate nanomaterials such as CNTs and is recommended for future studies.

Each technique used in the study is based on different principles with limitations to all. DLS is not an ideal technique for non-spherical suspensions as well as highly polydisperse systems. In such conditions, DCS is recommended because of high resolution, accuracy, and precision^40^. DCS can completely separate very narrow size distribution peaks that differ by as little as 3%. TEM provides direct visual images of the nanoparticles and is a great tool for the determination of aggregation, dispersion, size, and shape of the particles, but the technique requires sample drying which may lead to artifacts^41^. This can be eliminated by washing the grids with ultrapure water as discussed in step 4.5.3.

Amongst others, the methodology highlights some critical steps such as the type of vials used in the protocol, immersion depth, and position of the vials in the ultrasonic bath as well as the vial tweeter. Temperature control of the system during agitation is an important parameter. Frequent water changes in the ultrasonic bath and pulsed mode run in the case of vial tweeter are recommended to avoid any heat buildup during sonication, thus avoiding any sample alterations. The pre-wetting step for hydrophobic samples such as Zinc oxide helps in the dispersion of particles but this may induce some undesired changes. The sonication time and energy should be high enough to de-agglomerate the particles but not too much that it breaks the particles. The results indicate that agglomerate breakage is dependent on particle type.

Our findings highlight the importance of having a detailed dispersion protocol, as results show that key physico-chemical properties can potentially be altered during the sonication process, as governed by factors such as sonicator type, sonication duration time, and power output. Results have shown that sample integrity is potentially compromised at higher intensity agitation. Results show that CNTs are very sensitive to agitation, so breakages are highly likely to occur when sonication duration and strength are changed. Near to optimal settings for the dispersion of CNTs are between 2 - 15 min in the ultrasonic bath and only 2 min using the ultrasonic probe. However, the ultrasonication still may have caused some nanotube shortenings, which cannot be accurately quantified here. DLS may not be an ideal technique for the characterization of CNTs but it still can provide hydrodynamic diameter for nanotubes and this data could be informative of the differences in length distributions of CNTs amongst various samples^16^,^42^,^43^. Past studies demonstrate that the dispersion protocol of CNTs can be greatly enhanced by the addition of surfactants as the surfactant molecules are absorbed on the nanotube monolayer, thus providing a barrier to breakage due to sonication^35^,^44^. However, this cannot be compared directly to the present protocol as no surfactants are involved in this case. It is important to note that ensuring the length size distribution in the case of CNTs is very important, as the aspect ratio is often correlated with certain toxicological response. In contrast, CeO_2_ gave different results compared to the CNTs, in which prolonged sonication times using either ultrasonic bath or probe, lead to the formation of primary particles. The difference in findings between CNT and CeO2 cases highlights the importance to tailor dispersion protocols *e.g.*, optimize sonication time and power output, in accordance to starting material *i.e.*, type of nanomaterial powders. Every nanomaterial powder sample type is different, as there will be different degree of agglomeration within the powder itself. In certain instances, the de-agglomeration process has successfully resulted in the de-agglomeration up to primary particles level, as evident by the emergence of other shaped particles in the TEM images, which was not visible prior to the sonication step. The prolonged sonication resulted in the continuous breaking of cerium oxide agglomerates at different angles thus leading to multi-faceted particles.

In the case of commercially-bought aqueous sample of Ag NPs dispersions, our findings also emphasize the need for long term stability and uniformity assessment. There is a need to ensure that dispersions have been sufficiently characterized prior to use, especially in cases of long term storage. However, nanomaterials have a very short shelf life. They age with time and may behave differently after long term storage as compared to a freshly prepared dispersion.

The results here highlight the need for a harmonized strategy to identify an optimized protocol for different nanomaterials. The presented proposed strategy is to conduct different variations in the sonication method and to ensure that the dispersions at different time points are sufficiently characterized using complementary analytical methods. The importance on the use of a multi-method approach to characterize and monitor dispersion quality through time and different experimental conditions has been highlighted by past workers^45^. Although various methods for sonication have been presented to cater to specific nanomaterial dispersion in the study, potentially they can be used as the basis to disperse other metal and metal oxide nanomaterials (of similar surface properties) in water. However, having any change in either nanomaterial type or liquid medium requires the need to optimize the basic protocol, which can be done by careful adjustment of various factors *e.g.*, sonication time, strength, and sonicator type. Whatever protocol is chosen and identified as optimal, there is always a need to have a detailed report on the scheme and step-wise sequence of the sonication dispersion procedure. This is important to improve interpretability and comparability. One of the applications of this protocol is to facilitate data comparability amongst other labs leading to a harmonized and standardized approach for future studies. The current methodology and control parameters can be utilized for other dispersing mediums apart from water and comparisons can be drawn on a case by case basis.

## Disclosures

The authors have no competing financial interest. IK and EVJ jointly conceived the study with IK's design, performed the experiments, analyzed data, and prepared the manuscript. LJE and IR carried out the TEM imaging. SA, MLM, and MC supplied the CNTs, and rest of the co-authors discussed and commented on the manuscript at all stages and RT contributed towards the editing of the manuscript.
